# Artificial Intelligence in Dental Radiology: Between Technological Innovation and Clinical Responsibility

**DOI:** 10.21142/2523-2754-1402-2026-281

**Published:** 2026-04-04

**Authors:** Heraldo Luís Dias da Silveira

**Affiliations:** 1 Oral Radiology Department, Federal University of Rio Grande do Sul. Porto Alegre, Brazil. heraldo.silveira@ufrgs.br Universidade Federal do Rio Grande do Sul Oral Radiology Department Federal University of Rio Grande do Sul Porto Alegre Brazil heraldo.silveira@ufrgs.br

The integration of artificial intelligence (AI) within oral radiology signifies a fundamental shift in the interpretation of images and their subsequent application in clinical judgment. Algorithms leveraging deep learning have shown reliable efficacy in identifying caries, periapical lesions, root resorptions, and periodontal bone changes, frequently achieving sensitivity and specificity metrics that are either on par with, or in some instances, exceed those of human practitioners ^1^^-^^3^.

The rapid dissemination of these technologies demands a critical analysis that transcends mere performance validation. Radiological practice is not limited to identifying patterns in images, but involves clinical contextualization and the integration of multiple variables, which still represents a limitation of automated systems ^4^. In this sense, the risk of uncritical automation of diagnosis cannot be neglected.

Another relevant point concerns the origin of the data that feed the algorithms. AI models are highly dependent on the quality, diversity, and representativeness of their training datasets. The presence of biases can compromise the extrapolation of results, leading to diagnostic discrepancies in different populations ^5^. This limitation raises important ethical questions in healthcare.

Additionally, the incorporation of AI imposes regulatory and legal concerns that are still being consolidated. The definition of responsibility in cases of diagnostic error mediated by automated systems remains diffuse, requiring the construction of clear normative guidelines ^6^. The transparency of algorithms-often described as "black boxes" also presents an obstacle to their full clinical acceptance ^7^.

In the context of professional training, the emergence of these technologies demands a curricular reconfiguration that includes competencies in the critical interpretation of AI systems, understanding their limitations, potential biases, and ethical implications ^4^. Digital literacy, therefore, becomes essential to contemporary dental practice.

From a pragmatic perspective, AI should be understood as an instrument for expanding diagnostic capacity, capable of optimizing workflows and also reducing inter-examiner variability ^8^. However, its integration into clinical practice must occur judiciously, supported by robust evidence and aligned with the principles of evidence-based dentistry.

Consequently, the core concern transcends a binary acceptance or rejection of artificial intelligence; it centers on delineating the boundaries of its practical deployment. The trajectory of dental radiology will be determined by the capacity to judiciously and ethically merge human proficiency with technological capabilities. While innovation is a foregone conclusion, the imperative of responsibility is paramount.
